# Immune biomarkers for predicting response to adoptive cell transfer as cancer treatment

**DOI:** 10.1007/s00251-018-1083-1

**Published:** 2018-09-20

**Authors:** Ianthe A. E. M. van Belzen, Can Kesmir

**Affiliations:** Theoretical Biology and Bioinformatics, Utrecht University, Padualaan 8, 3584 CH Utrecht, The Netherlands

**Keywords:** Cancer, Adoptive cell transfer (ACT), Immunotherapy, Neoantigens, Biomarkers

## Abstract

Adoptive cell transfer (ACT) is a form of personalised immunotherapy which has shown promising results in metastasised cancer. For this treatment, autologous T lymphocytes are selected and stimulated in vitro before re-administration in large numbers. However, only a fraction of patients benefit from ACT, and it is not yet known what biomarkers can predict treatment outcome. In this review, we describe what tumour characteristics are associated with response to ACT. Based on the current knowledge, the best candidate biomarker for a good anti-tumour response seems to be a large number of neoantigens with a homogeneous distribution across the tumour in combination with sufficient MHC-I expression level. Additionally, it is necessary to be able to isolate a diverse population of T cells reactive to these neoantigens from tumour tissue or peripheral blood. Additional promising candidate biomarkers shared with other cancer immunotherapies are a large number of tumour-infiltrating cytotoxic and memory T cells, normal levels of glycolysis, and a pro-inflammatory cytokine profile within the tumour. Intense research in this field will hopefully result in identification of more biomarkers for cancers with low mutational load.

## Introduction

A cell needs to successively acquire characteristics via genetic modifications in order to become cancerous and escape the mechanisms that control cell proliferation and tissue integrity (Hanahan and Weinberg 2011). In order to mount an immune response against the cancerous cells, the genetic mutations should be visible to the immune cells. One possibility is the presentation of the mutated parts of the proteins on the cell surface. All cells with a nucleus present self-peptides on their surface in the context of class-I major histocompatibility complex (MHC), also known as human leukocyte antigen (HLA). T cells screen the peptides presented on MHC molecules for signs of “foreignness” (in case of an infection) or “alterations” from the healthy self-peptide repertoire. When a mutated peptide is presented on the cell surface, the immune system can recognise and clear cancerous cells. The peptide needs to have high affinity for the MHC molecule, to be recognised by specific T cell receptors (TCR) and form a stable complex in order to be immunogenic (Strønen et al. 2016). This high specificity-high affinity binding is thought to be a limiting factor in the antigen presentation process (Yewdell and Bennink 1999).

## The anti-tumour immune response and its bottlenecks

The cancer immunity cycle summarises how the immune response to cancer arises (Chen and Mellman 2013). When a tumour cell dies, cell debris is taken up by dendritic cells (DCs). The proteins in the debris are degraded into shorter fragments, resulting in a mixture of regular (wild type) and tumour-specific mutated peptides. The dendritic cells migrate to a nearby tumour-draining lymph node where they prime activation of T cells. Naive T cells are stimulated to mature if they recognise a presented antigen. Next, mature tumour-specific cytotoxic T cells (CTLs) migrate towards the tumour following a gradient of chemotactic signalling molecules, eventually infiltrating the tumour micro-environment. CTLs recognise their target cell by specific binding of their receptor to their cognate epitope: tumour cells expressing the same MHC class I-peptide complex as the tumour associated peptide presented by the dendritic cell in the lymph node. This triggers CTLs to release effector molecules which kills their target tumour cells.

Unfortunately, anti-tumour immune responses are not always successful, and evasion by the cancer cells is one of the hallmarks of the cancer pathology (Hanahan and Weinberg 2011). Three prominent issues or “bottlenecks” are identified so far (Van De Ven and Borst 2015). Firstly, tumour cells can evade the immune response by avoiding presentation of antigens which can distinguish cancerous cells from self. Ongoing selection pressure in the tumour for this evasion results in cancerous cells which are increasingly hard to clear (Vesely et al. 2011). Secondly, the effector function of CTLs is dependent on priming by activated DCs. However, the molecules necessary to activate pattern recognition receptors on DCs are often not present in tumour material (Van De Ven and Borst 2015). When a TCR-MHC interaction takes place with an unactivated (immature) DC, peripheral tolerance can be induced for that clone: T cells are rendered unresponsive (anergic) or differentiate into immunosuppressive regulatory T cells (Tregs). Third, the tumour micro-environment is often immunosuppressive. This is characterised by the presence of inhibitory cytokines and Tregs which suppress the cytotoxic activity of CTLs, and through expression of inhibitory ligands such as programmed death-ligand 1 (PD-L1) (Fridman et al. 2012).

## Immunotherapies for treatment of cancer

Re-establishing immunological control of the tumour is the main objective of cancer immunotherapy. Therefore, the bottlenecks mentioned above can also be considered as goals for treatment development. The immunotherapies can be classified into three main groups: non-specific immunomodulation, cancer vaccines and adoptive cell transfer. The non-specific immunomodulation therapies aim at strengthening the general CTL response irrespective of the specificity for tumour, like the administration of interleukin-2 (Rosenberg et al. 2008). This category also includes immune checkpoint blockade treatments which make use of antibodies against inhibitory molecules, for example cytotoxic T lymphocyte associated 4 (CTLA-4) (Snyder et al. 2014) and programmed death-ligand 1 (PD-L1) (Rizvi et al. 2015). Cancer vaccines contain tumour-specific proteins or peptides in conjunction with adjuvant in order to elicit the expansion of tumour-specific CTLs. It has been demonstrated that such immunisations can result in an improved T cell priming and activation by DCs (Rosenberg et al. 2008). However, a large number of tumour-specific CTLs is not always associated with tumour regression, possibly due to immunosuppressive properties of the tumour micro-environment (Chen and Mellman 2013).

Adoptive cell transfer (ACT) is a personalised cancer immunotherapy where lymphocytes acquired from the patient are used. These lymphocytes are selected based on how well they recognise the tumour and are restimulated in vitro, in order to revert them back to efficient immune cells. Depletion of lymphocytes in the patient prior to readministration of specific lymphocytes considerably improves the efficacy of ACT (Goff et al. 2016), probably due to removal of suppressive immune cells. Eventually, a large number of T cells with more efficient phenotype are readministered to the patient (Rosenberg and Restifo 2015).

One of the cancer types which promises success with ACT is metastatic melanoma (Rosenberg and Restifo 2015; Tran et al. 2017), because tumour-infiltrating lymphocytes (TILs) with a good anti-tumour reactivity can often be isolated from resected tumour tissue. Moreover, the mutational load in melanoma is very high (Lawrence et al. 2013), which results in a larger number of immune targets for ACT. In many other cancer types, the mutational load is lower and TILs are not always easily accessible. Peripheral blood lymphocytes (PBLs) are used as alternatives to TILs, together with procedures to enrich for tumour-specific T cells. It is also possible to genetically engineer PBLs with high-affinity receptors (Morgan et al. 2013; Parkhurst et al. 2011). However promising, there are still issues regarding the application of ACT in clinical practice. Its use is currently limited to only some types of cancer, and it is unclear why some patients respond well and others do not. There is a high need for reliable biomarkers which make it possible to select the patients who could benefit from this novel personalised treatment.

For a characteristic to be a useful biomarker, it needs to satisfy several criteria (Willis and Lord 2015). Firstly, the characteristic should be able to correctly classify the patients according to treatment outcomes and ideally reflect an underlying mechanism of disease. It needs to be practical to perform and be measurable in a high-throughput assay that is fast and not too costly. It is also important to standardise this procedure, so the results can be compared between patients and different institutes. Finally, the biomarker should be validated in multiple studies involving large groups of patients.

**Fig. 1:**
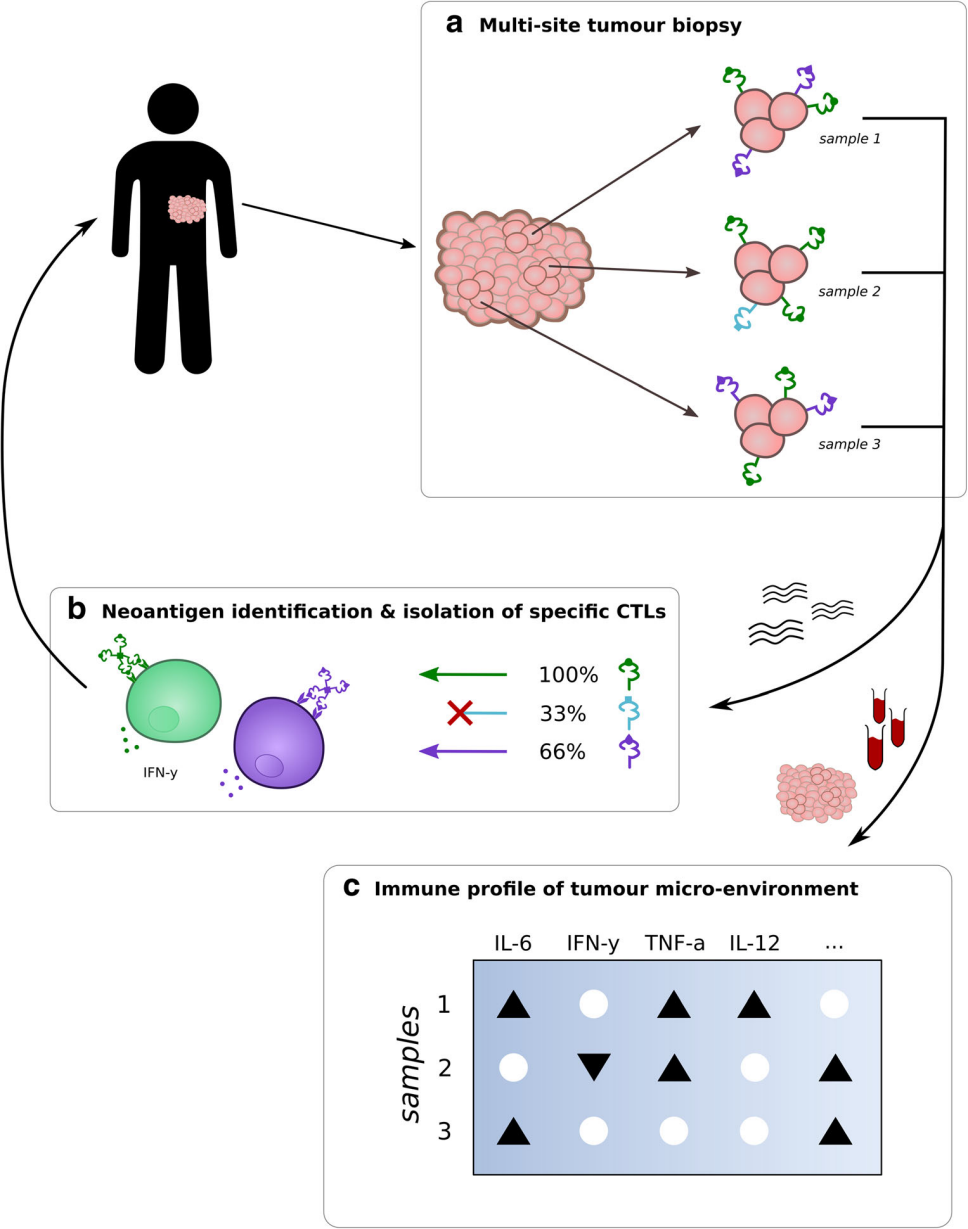
Overview of the suggested biomarkers: (1) the presence of neoantigens with homogenous distribution in the tumour for which responsive T cells can be isolated in vitro for adoptive cell transfer (ACT) therapy (A and B), (2) immunological characterisation of the tumour microenvironment to determine whether additional intervention is necessary (C). A) multiple biopsies should be taken from the tumour, each being likely to have a different neoantigen presentation signature due to heterogeneity in the tumour. The biopsies are subjected to whole exome sequencing (WES). B) Neoantigens and their distribution in the tumour can be identified based on the WES reads of the samples. One neoantigen (green, round) is present in all biopsies, one is present in only a small group of cells (blue, square) and one in a majority (purple, diamond). It is recommended to use multiple targets for ACT with a homogeneous distribution within the tumour to prevent immune escape. Next, MHC tetramers (indicated as groups of four MHC molecules in the figure) are used to isolate T cells specific for the selected neoantigens. These CTLs should also have an effector phenotype characterised by e.g. secretion of IFN-y. C) an immune profile is determined based on the different biopsies and the patient’s blood. The balance between immunostimulatory and -suppressive factors in the TME influences the effector phenotype of T cells and is predictive for the response to immunotherapy. In general, proinflammatory cytokines like IL-2, IL-12 and IFN-gamma are associated with improved outcome, as well as M1 macrophages, CD8+, D4+ and memory T cells, while the cytokines IL-6, IL-10 and TGF-B, as well as lactic acid and MDSCs, are associated with less response to treatment. The effect of Tregs on anti-tumour immune responses seems to differ between cancer types. Only a few of these factors are depicted in the figure

For adoptive T cell transfer, the most promising biomarkers so far are related to the interaction of the patient’s immune system with the tumour, we review the products of most of the immune screening methods and propose candidate biomarkers for predicting response to ACT treatment (Fig. 1).

## Potential biomarkers in peripheral blood

Recent advances in immune monitoring methods have made it possible to use peripheral blood for characterisation of the patient’s immune profile (Whiteside 2013). Specific blood-borne immunomodulatory factors can serve as potential biomarkers. Cytokines, for example, which are known for their regulatory role in immune responses and their specific signalling functions between immune cells, are good candidates. A meta-analysis by Lippiz considered cytokine profiles for 13 types of cancer, amongst others colorectal cancer, melanoma and lung cancer. High serum levels of the “immunosuppressive” cytokines in blood, like interleukin (IL)-6 and IL-10, are found to be correlated with poor prognosis, cancer stage and disease progression (Lippitz 2013). However, especially, cytokine levels in the tumour micro-environment are important for shaping the anti-tumour response (Fridman et al. 2012). Unfortunately, the cytokine levels in the blood do not always reflect the cytokine concentration in the tumour environment.

One of the immunosuppressive mechanisms used by tumours is the secretion of apoptosis inducing factors for CTLs, which can sometimes also be detected in the blood. Hence, the frequency with which CTLs in the periphery undergo apoptosis can be a measure indicating the strength of immunosuppression. Apoptosis of CTLs can be induced by ligands such as FasL or TNF-related apoptosis-inducing ligand (TRAIL) that are either secreted or present in tumour-derived exosomes (Huber et al. 2005). These ligands contribute to rapid turnover and lymphocyte depletion. In addition, chronic inflammation has been found to contribute to further growth of some types of cancer, e.g., colorectal cancer and inflammatory bowel disease, and cervical cancer and infection with HPV (Mantovani et al. 2008). A marker of chronic inflammation is a high neutrophil-to-lymphocyte ratio, which has been associated with poor clinical outcome in various types of cancer especially together with a low total lymphocyte cell count (Perisanidis et al. 2013; Whiteside 2013).

More recent studies have identified immunological signatures that can be determined with routine blood analysis and are able to predict responses to checkpoint blockade immunotherapy, such as antibodies against CTLA-4 and PD-L1. Martens et al. identified peripheral biomarkers in 209 advanced melanoma patients that were associated to clinical outcome after anti-CTLA-4 treatment (ipilimumab) (Martens et al. 2016). A signature consisting of a low absolute monocyte count, myeloid-derived suppressor cells (MDSCs) and lactate dehydrogenase as well as high absolute eosinophil counts, relative lymphocyte count and Tregs is significantly associated with overall survival and treatment response. Tanizaki et al. found similar biomarkers in an analysis of 134 non-small cell lung cancer (NSCLC) patients (Tanizaki et al. 2018). A low absolute neutrophil count, high absolute eosinophil count and high absolute lymphocyte count were associated with survival after anti-PD-1 treatment (nivolumab). It is not yet clear whether peripheral lymphocyte population counts can be used as biomarker for ACT specifically. Although some of the biomarkers for anti-CTLA-4 or anti-PD-1 are likely to be indicative of the potential to mount an effective CTL response, e.g., high eosinophil or lymphocyte counts and low MDSC count, others are probably treatment specific, such as the high Treg number being predictive for a response to anti-CTLA-4 treatment. Hence, while these peripheral measurements are easily accessible with regular blood tests, further research is needed specifically for ACT to reliably use them for prediction of treatment outcome to cell transfer.

Intuitively, the most promising peripheral blood biomarker for ACT would be the number of tumour-specific T cells. Isolating tumour-specific CTLs for adoptive cell transfer is done preferably from resected tumour tissue (Rosenberg et al. 2008). However, this is not possible for all cancer types, and the peripheral T cell pool can be an alternative source, although it contains a lower fraction of tumour-specific cells. The detection of tumour-specific T cells needs to be done in a high-throughput fashion in order to be clinically useful. At the moment, MHC multimer technology using multiple fluorochromes and combinatorial encoding seems to be the tool that allows for parallel detection of largest T cell populations recognising different tumour-specific antigens (Hadrup et al. 2009). Recently, Cohen et al. were the first to demonstrate the usage of MHC tetramers for isolation of tumour-specific T cells from peripheral blood prior to expansion induced by immunotherapy (Cohen et al. 2015). For five out of eight patients, a total of nine epitopes with tumour-specific mutations was predicted. CTLs could be isolated from blood for eight of these epitopes, their frequencies estimated between 0.002 and 0.4%. In order to confirm that CTLs are responsive to specific epitopes, their effector cytokine production is measured, e.g. with interferon-γ ELISPOT technology. This assay is relatively easy to perform as it does not involve flow cytometry, and it has already been associated with clinical outcome (Schaefer et al. 2012). Another promising method for isolating CTLs that respond to patient-specific cancer mutations is described by Gros et al. They were able to successfully use the expression of PD-1 as biomarker for tumour-specific CTLs in peripheral blood of three out of four melanoma patients (Gros et al. 2016). These patients showed PD-1 expression in 36% of their tumour-infiltrating CTLs and in 4% of peripheral cells, of which only a small fraction was actually tumour-specific; hence, sufficient PD-1 expression is probably necessary for using this molecule as additional biomarker. Finally, Simoni et al. showed that CD8+ TILs are not only specific for tumour antigens, but also by being “bystander” cells, they recognise a wide range of epitopes (such as those from Epstein-Barr virus, human cytomegalovirus or influenza virus) (Simoni et al. 2018). These bystander CD8+ TILs lack CD39 expression, while tumour-specific TILS have high CD39 expression, suggesting that CD39 can also be used to enrich tumour-specific cells during ACT. Although the intuitive link between the presence of peripheral tumour-specific CTLs to ACT response needs to be demonstrated, isolation of a diverse T cell population with effective phenotype either from the tumour or from blood is a prerequisite for successful cell transfer therapy (Schumacher, personal communication).

For checkpoint-blockade immunotherapy, specific T cell populations can be indicative of response to the intervention. Kamphorst et al. characterised peripheral blood mononuclear cells (PBMCs) before and during treatment of 29 NSCLC patients with anti-PD-1 or anti-PD-L1 antibodies (Kamphorst et al. 2017). Flow cytometry was used to distinguish the markers CD4+, CD8+, FoxP3+ and Ki-67+ on T cells, where FoxP3+ was used as to distinguish Tregs and Ki-67+ as indication of an effector phenotype. In 70% of the patients, an increase in PD-1+ CD8+ Ki-67+ T cells was observed after treatment. Furthermore, patients with clinical benefit and progressive disease could be distinguished based on an early increase of this T cell population, although no significance could be achieved in this small cohort. No differences were observed in pre-treatment state of the patients, other than having lower lymphocyte counts and less naive CD8+ T cells compared to healthy controls. Hence, whilst monitoring of peripheral T cell populations can provide insight in response to treatment, it was not possible to predict this response beforehand. However, characteristics of the tumour-infiltrating T cell population, names as immunoscore (Galon et al. 2012; see section “Influence of the tumour micro-environment”), are currently undergoing extensive research and validation for use as a general cancer biomarker.

## Tumour-specific antigens recognised by CTLs

Cancer immunosurveillance is thought to be an important extrinsic tumour suppressor, in which immune cells are able to recognise and clear aberrant cells—often before they pose a real danger (Vesely et al. 2011).

While both the innate and adaptive immune response play a role in immunosurveillance, the latter is dominant in controlling the growth of an established tumour (Koebel et al. 2007). In order to accomplish this, T cells should be able to specifically bind epitopes that are only present on the surface of cancer cells and not on healthy cells.

Antigens which can help T cells to distinguish tumour cells from healthy cells are divided in two classes: tumour-associated antigens (TAAs), which can also be present in healthy cells, but often expressed in much higher levels by tumour cells; and tumour-specific antigens (TSAs) which are unique for tumours (Kelderman and Kvistborg 2016). TAAs are derived from proteins that are normally expressed in germline cells or differentiation antigens specific to a cell lineage. TSAs, on the other hand, are formed in tumour cells as a result of accumulated mutations, insertions, deletions or a viral infection. A special category of TSAs, neoantigens, is the missense mutations in proteins that are presented in context of the patient’s MHC molecules. Tumour-specific antigens are the most desired targets for ACT, because they are not expressed in healthy tissue or in the thymus (Kelderman and Kvistborg 2016). Due to the high specificity of TCRs, T cells recognising a neoantigen are not expected to be self-reactive. Thus, they are not likely to cause auto-immunity or be deleted during negative selection.

Viruses like human papillomavirus (HPV) and Epstein-Barr virus (EBV) have the potential to cause cancer. As these viral antigens are rather different than self-peptides, they form excellent targets for ACT. This was also illustrated with successful ACT trials using CTLs selected for their reactivity to these viruses (Table 1). In a study for metastatic cervical cancer using HPV reactive TILs, two of nine patients displayed a complete response and one patient a partial response (Stevanović et al. 2015). In 11 of 13 lymphoma patients, ACT treatment with EBV reactive CTLs resulted in complete remission (Heslop et al. 2010). An advantage of viral antigens compared to neoantigens is their more homogeneous presentation in the tumour and higher immunogenicity. Nevertheless, targeting neoantigens with ACT makes it applicable to a broader range of cancer types.

**Table 1:** Overview of reviewed clinical trials

ACT variant^a^	Cancer type	*N*	%OR	Outcome (CR/PR/SD/AD)	Reference
TILs	Melanoma	93	56	19 CR, 33 PR	(Rosenberg et al. 2011)
TILs	Melanoma	101	54	23 CR, 30 PR	(Goff et al. 2016)
TILs, single neo-epitope enriched	Gastro-intestinal	4	50	1CR, 1 PR	(Tran et al. 2015)
TILs reactive to HPV	Cervical	9	33	2 CR, 1 PR	(Stevanović et al. 2015)
EBV-specific CTLs from cell lines	Lymphoma	13	85	11 CR	(Heslop et al. 2010)
DC vaccine primed PBLs	Ovarian	3	66	1 CR, 1 SD	(Kandalaft et al. 2013)
HER2/neu vaccine primed PBLs	HER2+ breast and ovarian	7	43	3 PR	(Disis et al. 2014)
PBLs w/ engin. CEA reactive TCR	colon	3	33	1 PR 3 AD severe inflammatory colitis	(Parkhurst et al. 2011)
PBLs w/ engin. MAGE-A3 reactive TCR	MAGE-A3/12+ tumours	9	55	1 CR, 4 PR 3 AD neurotoxicity (two deaths)	(Morgan et al. 2013)
PBLs w/ engin. NY-ESO-1 reactive TCR	Melanoma	20	55	4 CR, 7 PR	(Robbins et al. 2015)
PBLs w/ engin. NY-ESO-1 reactive TCR	Synovial cell sarcoma	18	61	1 CR, 10 PR	(Robbins et al. 2015)

TAAs: carcinoembryonic antigen (CEA) New York esophageal squamous cell carcinoma-1 (NY-ESO-1), human epidermal growth factor receptor 2 (HER2/neu), and melanoma-associated antigen (MAGE). Epstein-Barr virus (EBV) human papillomavirus (HPV). RECIST criteria were used for describing outcome: complete response (CR), partial response (PR), objective response (OR) both CR and PR. Stable disease (SD), adverse effects (AD). We have chosen not to indicate the patients that eventually progress to disease, as this heavily depends on the follow-up time of the cohorts

^a^Tumour infiltrating lymphocyte (TIL), peripheral blood lymphocyte (PBL), cytotoxic T lymphocyte (CTL), T cell receptor (TCR), tumour-associated antigen (TAA), and dendritic cell (DC). *N* is the number of patients

## Neoantigens and mutational load

The identification of neoantigens on a large scale requires whole exome sequencing (WES), in combination with algorithms predicting possible immunogenic mutations (van Rooij et al. 2013). Subsequently, screening measures like MHC tetramers or neoantigen-pulsed APCs can be used to check for the presence of neoantigen-specific T cells (Schumacher and Schreiber 2015). With the availability of large patient cohorts and sequence facilities, efforts were made to identify a set of shared mutations which could provide a selective advantage to the tumour. Contrary to expectations, meta-analysis using data from The Cancer Genome Atlas (TCGA) found that neoantigens are almost always unique for the patient, with a maximum of two to three patients sharing an epitope (Nguyen et al. 2016). Similar results were obtained in smaller clinical trials in melanoma (Tran et al. 2015; Linnemann et al. 2015) and a larger study in colon cancer (Angelova et al. 2015). This underlines the importance of determining patient-specific targets for ACT with the use of tumour sequencing in conjunction with immunogenicity predictions such as NetMHCpan (Nielsen and Andreatta 2016) and/or other algorithms (Lee et al. 2018; Marty et al. 2017).

The frequency of mutations can differ up to 1000-fold between different types of cancer as well as between patients of the same type (Lawrence et al. 2013). Melanoma and NSCLC have the highest mutation frequencies (100/Mb), whilst e.g. clear cell renal cell cancer (ccRCC) and breast cancer are types with a more moderate mutation frequency (1/Mb) (Lawrence et al. 2013). Intuitively, cancer types with a high mutation frequency can be expected to have more neoantigens. In agreement with this, a positive correlation has been found between mutational load, predicted number of neoantigens and response to immune checkpoint blockade therapy for melanoma and NSCLC (Snyder et al. 2014; Rizvi et al. 2015; Van Allen et al. 2015). Although ccRCC appears to be sensitive to immune therapies, this is less well established (Matsushita et al. 2016). On the other hand, a patient with breast cancer was successfully treated with autologous CTLs against four neoantigens, leading to durable regression (Zacharakis et al. 2018). It is suggested that the success of immunotherapy in melanoma and NSCLC is due to the large number of neoantigen-specific CTLs that are stimulated by the intervention (Schumacher and Schreiber 2015).

It is also important to consider the degree of clonality with respect to the presentation of neoantigens in the tumour. In a recent comparison of data on mutational heterogeneity within and across different cancer types, melanoma and lung cancer were found to be quite homogeneous and bearing a large amount of clonal mutations (McGranahan and Swanton 2017). It is likely that the high homogeneous mutational load of skin and lung cancers is influenced by pre-cancer exposure to mutagens such as ultraviolet light and tobacco (Alexandrov et al. 2013). Additionally, these cancers were found to be more sensitive to immune checkpoint blockade treatment if they were enriched in clonal neoantigens, combining the neoantigen burden with heterogeneity improved the significance of the association (McGranahan et al. 2016). In colon cancer, more homogeneous tumours also showed improved immune responses and better prognosis (Angelova et al. 2015). Apparently, within-tumour heterogeneity is widespread, and this highlights the importance of sequencing multiple regions of the tumour and considering clonality of neoantigens in the selection of suitable targets for ACT.

## Immune escape: immunoediting and downregulation of MHC-I

Recognition of neoantigens by CTLs depends not only on the mutation load but also on the presentation of mutated peptides in the context of a MHC class-I molecule of the patient. There is a large variety in the binding motifs of HLA molecules and therefore the set of presented peptides differs a lot (Robinson et al. 2014). For example, the proto-oncogene multiple myeloma SET was presented by only three different MHC molecules (Walz et al. 2015). Therefore, direct associations between specific HLA molecules and treatment response are difficult to establish with significance (Szender et al. 2016; Alcoceba et al. 2013).

Which antigens are presented on the tumour cell surface is substantially influenced by interactions with the immune system (Marty et al. 2017; Schumacher and Schreiber 2015). The immune system eliminates the most immunogenic tumour cells, and therefore those that can best evade the immune response have a proliferative advantage. Vesely et al. formulated a cancer immunoediting model which summarises this interaction (Vesely et al. 2011) using three phases: elimination, equilibrium and escape. In the elimination phase, the immune system is able to remove cancerous cells before a malignant tumour is formed. The equilibrium phase ensues when elimination of all tumour cells is no longer possible. Tumours can be dormant for a long time in this phase. The opposing forces of tumour proliferation and destruction are balanced, whilst immunoediting continues and the tumour microenvironment becomes increasingly immunosuppressive. Eventually, tumour cells emerge for which it is difficult to mount any efficient immune response, and the tumour escapes from immune surveillance. Marty et al. recently showed the first evidence of immunoediting in a large-scale analysis from TCGA data considering the patient’s HLA-I molecules and 1018 recurrent mutations in known driver genes (Marty et al. 2017). Which oncogenic mutations can accumulate without being detected by the immune system depends on the patient’s HLA genotype, as recurrent mutations, i.e., occurring in several patients, were biased for poor presentation by common HLA alleles (Marty et al. 2017).

Decreased cell surface presentation of immunogenic MHC-I-peptide complexes is one of the most effective escapes from the immune response (Chen and Mellman 2013; Vesely et al. 2011). The downregulation of MHC-I molecules was associated with poor prognosis in multiple studies (Perea et al. 2017; Mariya et al. 2014; Zeestraten et al. 2014). Perea et al. found that MHC-I negative lung tumours showed less CTL infiltration and a different composition of TILs, which was associated with poor outcome. About half of the lung tumours completely lost MHC-I expression, and the other half had a significant fraction of cells which downregulated MHC-I. The loss of expression was due to silencing of the HLA genes or β2-microglobulin (B2M), which is a necessary component of the MHC-I-peptide complex (Perea et al. 2017). A similar study conducted in epithelial ovarian cancer showed that expression of MHC-I and low CTL infiltration was inversely correlated with survival (Mariya et al. 2014). Zeestraten et al. conducted a multivariate analysis on 285 patients with colon cancer and were able to distinguish three different immune phenotypes which were correlated with overall survival and disease-free prognosis (Zeestraten et al. 2014). When MHC-I expression was considered as a separate predictor, patients with a complete loss had a significantly better prognosis compared to those with downregulation or regular expression. This is not surprising, as loss of MHC-I would make a tumour prone to clearance by NK cells (Khong and Restifo 2002). However, one could also expect NK cell recognition as a result of substantial downregulation. One possible explanation of why this does not seem to happen frequently could be the downregulation of NK cell activating ligands on tumour cells (Salih et al. 2002).

Alternatively, neoantigens can also be lost as a result of genomic alterations or Darwinian selection in case of subclonal mutations. Anagnostou et al. studied the changes in neoantigen landscape in four NSCLC patients which had developed acquired resistance to checkpoint blockade treatment after initial response, an effect that is exemplary for immunoediting in clinical practice (Anagnostou et al. 2017). In this study, neoantigens were identified using tumour WES in conjunction with immunogenicity prediction of mutated peptides by NetMHCpan and the patient’s HLA genotype. Comparing pre- and post-treatment tumour samples revealed two novel mechanisms of neoantigen loss: deletion of chromosomal regions containing the mutated peptide and elimination of tumour cells which presented the neoantigen (Anagnostou et al. 2017). The maintained mutations were less likely to be immunogenic, and not in known tumour suppressor or antigen-presentation associated genes. In these patients with acquired resistance, 7 to 18 neoantigens were lost and it was shown that these lost epitopes could elicit T cell expansion within the autologous population (Anagnostou et al. 2017).

## Using neoantigen presentation as biomarker

The combination of the number of missense mutations and MHC-I expression would be a robust and easy measurable biomarker. This would require tumour WES and immunogenicity predictions based on the patient’s HLA-I alleles and their binding affinity with the mutated peptide, such as was done by Marty et al. and Aganostou et al. Earlier studies also underline the importance of combining MHC-I expression and the mutational load. In a meta-analysis across cancer types, the number of neoantigens was correlated with increased survival and CTL infiltration only if they were predicted to be immunogenic (Brown et al. 2014). Brown et al. used three criteria to filter mutated peptides on high immunogenicity: elevated expression of the mutated gene, elevated expression of HLA-A and high predicted binding affinity (IC50 value 500 nM or less) for the MHC-I-peptide complex. For clear cell renal cell carcinoma, a type of cancer with moderate mutation burden, it was already established that the number of mutations needs to be combined with MHC-I expression to achieve the best prognostic value (Matsushita et al. 2016). In addition, the heterogeneity of the tumour should be considered. Relatively homogeneous tumours are associated with higher overall survival, more neoantigens and immune cell infiltration, while the heterogeneous tumours have more potential for escaping the anti-tumour immune responses (Morgan et al. 2013; Angelova et al. 2015). More specifically, tumours with a high burden of clonal neoantigens were found to have the best response to immunotherapy (McGranahan et al. 2016).

Concluding, tumour recognition can be a bottleneck in spite of novel mutations, as a sufficient level of MHC-I-peptide complexes is required for the anti-tumour CTLs to be effective. In order to prevent an easy escape by loss of presentation of the targeted neoantigen, it is important to administrate a diverse set of CTLs with multiple targets (Schumacher and Schreiber 2015). Therefore, for adoptive cell transfer, it is beneficial to have multiple neoantigens available as target with sufficient cell surface presentation, together covering the entire tumour and thus prevent immune escape.

## Targeting tumours with low mutational loads

Tumours with low mutational load can have too little neoantigens to target. As an alternative to neoantigens in such tumours, TAAs can be used, which are proteins overexpressed in the tumour relative to healthy tissue. TCRs specific for TAAs are often removed by negative selection during T cell maturation. Clinical trials using modified high-affinity receptors to target TAAs vary in their success of attacking solid tumours and severity of side-effects due to auto-immunity (Parkhurst et al. 2011; Morgan et al. 2013; Robbins et al. 2015). As it is not possible to precisely characterise the tissue distribution of these antigens, using TAAs with engineered TCRs come with a high risk for off-target effects. This is illustrated by clinical trials targeting various TAAs (Table 1). On the other hand, for B cell lymphoma and leukaemia, an ACT treatment with autologous T cells engineered with a chimeric antigen receptor (CAR) against the B cell marker CD19 has shown high response rates and is recently approved by the FDA (Maude et al. 2018). However, the success of CAR T cell treatment has so far been limited to haematological cancers, and it is challenging to apply to solid tumours due to a.o. the heterogeneous distribution of antigens (Mirzaei et al. 2017).

In the first ACT for metastatic colon cancer targeting TAAs, modified cells were administered with a high-avidity TCR specific for carcinoembryonic antigen (CEA) (Parkhurst et al. 2011). CEA is often overexpressed in colorectal cancer cells compared to healthy adult cells, together with elevated levels in the patient’s blood. One of the three patients participating in this study showed a partial response, and all displayed a decrease of CEA level in the blood. However, all patients developed severe inflammatory colitis. This adverse effect was probably caused by CTLs responding to CEA-derived antigens on the healthy colon (Parkhurst et al. 2011). Another interesting TAA to target is melanoma-associated antigen (MAGE) which is present in about 30% of all epithelial cancers and suppresses the activity of tumour-suppressor gene p53 (Monte et al. 2006). An ACT trial targeting MAGE was conducted in nine patients having MAGE-positive tumours (Morgan et al. 2013). The results were unanticipated: While five patients had objective responses, three suffered severe neurotoxicities of which two resulted lethal. Retrospective analysis showed that MAGE was also expressed in the brain, a finding previously undocumented. These shocking results demonstrate how severe “on-target/off-tumour toxicities” can be. On the other hand, no adverse effects were reported for a trial with modified receptors specific for New York esophageal squamous cell carcinoma-1 (NY-ESO-1) (Robbins et al. 2015). Objective responses were recorded for 55% and 61% of the melanoma and synovial cell sarcoma patients, respectively.

A large study in colorectal cancer using 598 samples from TCGA considered amongst others the mutational load, type of mutations, infiltration by immune cells and cytokine profile (Angelova et al. 2015). Whilst the number and expression of cancer-germline antigens was similar between tumours, the neoantigen burden was very diverse. Evolution of the antigen landscape and intratumoral immune profile was found to be intertwined and directed towards immune evasion, with increased immunosuppressive factors and decreasing neoantigen frequency. For tumours with a low mutational load, homogeneous tumours had a better prognosis, whilst hypermutated tumours seemed less susceptible to immune editing, and effective immune responses were also observed in spite of heterogeneity (Angelova et al. 2015).

The improved immune detection of hypermutating tumours could be leveraged for types of cancer which often have a low mutation load. Germano et al. recently demonstrated this in a mouse model where inactivation of DNA mismatch repair mechanisms induced a hypermutator phenotype, resulting in increased neoantigen burden (Germano et al. 2017). While promising, this could also lead to complications such as inducing carcinogenic mutations in healthy tissue (Raj and Patil 2018). Given all these results, for cancer types with a low mutational load, immunotherapy in general seems to be a less favourable option due to the difficulty of immune recognition of cancerous cells.

## Influence of the tumour micro-environment

Another important predictor of disease outcome in cancer is the immune contexture. This concept was formulated to describe the location, density and phenotype of immune cell populations in the tumour micro-environment (TME) (Fridman et al. 2012). Especially the number of tumour infiltrating cytotoxic and memory T cells was found to have a strong correlation with survival in colorectal cancer (Mlecnik et al. 2016). Galon et al. formalised the count and location of these lymphocyte populations into an immunoscore and proposed to use this measure as an additional measure to classify cancers and the probability of success of immunotherapy (Galon et al. 2012). The immunoscore combines the presence of two T lymphocyte populations and their infiltration into the tumour core and its invasive margin. The number of T cells in both regions is counted by immunohistochemistry using markers for CD8+ cytotoxic and CD45RO+ memory T cells, or alternatively CD3+ since this is a more robust measurement than CD45RO+. A high immunoscore (I4) is attributed to patients which have high densities of these lymphocyte populations in both regions, and a low immunoscore (I0) corresponds to when the densities are low. One of the first illustrations of the prognostic value of the immunoscore was a study involving patients with an early stage of colorectal cancer (Pagès et al. 2009). Disease recurrence after surgery was increased in patients with a low immunoscore compared to a high immunoscore: After 5 years, 72% of the I0 patients had experienced relapse compared to 5% of the I4 patients. These findings were confirmed by a larger study recently on colorectal cancer patients in varying stages of the disease (Pagès et al. 2018). Also for other cancer types, such as gastric cancer and melanoma, the immunoscore has been a good predictor of the immunotherapy success (Jiang et al. 2018; Galon et al. 2017). However, the immunoscore by itself is not sufficient for making treatment predictions, because interactions with other immune cells and cytokines influence the effector phenotype of CTLs and memory T cells.

Anti-tumour T cells can be downregulated by immunosuppressive factors in the tumour micro-environment, which is also a mechanism of immune escape (Vesely et al. 2011). An increase of cytotoxic T lymphocytes and their effector molecules is observed to happen in conjunction with an increase in inhibitory cytokines and regulatory T cells in multiple studies (Matsushita et al. 2016; Perea et al. 2017; Parkhurst et al. 2011; Brown et al. 2014). In the meta-analysis by Brown et al., half of the tumours with predicted immunogenic neoantigens had increased expression not only of CTL marker CD8A but also of immunosuppressive markers such as CTLA-4 and PD-1 (Brown et al. 2014). The results of Matsushita et al. confirm this, as they found increased expression of CTLA-4, PD-1 and ligands of PD-1 in tumours classified as having high CTL infiltration. This result might explain why patient survival in this group of tumours with high CD8A and CTL effector molecules such as granzymes and perforin is not increased (Matsushita et al. 2016). Therefore, we suggest that next to the immunoscore, immunosuppressive factors in the TME need to be taken into consideration in order to invoke an efficient anti-tumour response by using ACT.

## Varying contributions of T helper cells

Until now, we focused on CD8+ cytotoxic T cells, but CD4+ T helper cells also have an important contribution in shaping anti-tumour immune responses. CD4s can even induce apoptosis of tumour cells by secreting cytotoxic granules or stimulating death ligands (Ahrends and Borst 2018). CD4+ T cells recognise peptides in the context of MHC class-II molecules, which are presented by specialised APCs, such as dendritic cells or macrophages. The role of type-1 helper cells (TH1) is to stimulate the activation and differentiation of CTLs in the lymph node. Additionally, in the TME, they support the effector phenotype of CTLs by secreting cytokines such as IL-2, IL-12, IFN-γ and tumour necrosis factor α (TNF-α). Both the presence of TH1 cells and their pro-inflammatory cytokines have a positive influence on the clinical outcome in cancer patients (Fridman et al. 2012; Mantovani et al. 2008). For the type-2 helper (TH2) population, this is less clear as correlations between tumour infiltration and prognosis differ between cancer types. However, the characteristic TH2 cytokines (IL-4, IL-5, IL-6, IL-10 and transforming growth factor β (TGF-β)) are mostly immunosuppressive and associated with poor outcome (Lippitz 2013).

The importance of CD4+ cells for establishment of an anti-cancer immune response is illustrated by two clinical trials with CD4+ cells. A patient with metastatic epithelial cancer was treated twice with infusion of neoantigen-specific TH1 cells and experienced a regression both times (Tran et al. 2014). Another study looked at the frequency of CD4+ cells amongst melanoma TILs by using an IFN-γ assay combined with an anti-HLA class-II antibody (Friedman et al. 2012). Approximately 20% of the responding cells were blocked by the antibody, indicating that they should be CD4+ cells. More importantly, it was shown that treatment with CD4+ TILs can mediate regression in a patient with advanced metastatic melanoma. This was unfortunately followed by disease progression of tumour cells which had downregulated the recognised HLA class-II molecule.

The role of TH17 cells in cancer remains elusive, as they have been reported to have a positive influence on prognosis for some types of cancer and a negative one for others (Fridman et al. 2012). In mice, IL-17, the main cytokine produced by TH17 cells, was found to increase proliferation of cancer cells in immunodeficient individuals via chronic inflammation, but support the anti-tumour response in immunocompetent animals (Wilke et al. 2011). In humans, TH17 cells seem to improve the anti-tumour response, possibly due to stimulation of TH1 cells and CTLs. However, the cytokine IL-17 is associated with poor outcome, most likely due to the recruitment of neutrophils. As other cells also produce IL-17 including neutrophils (Punt et al. 2015), it remains hard to produce a solid conclusion based on these results.

Immunosuppressive, self-reactive regulatory T cells (Tregs) help to prevent autoimmunity and maintain peripheral tolerance. Tregs carry TCRs specific for self-peptides and can suppress the proliferation of naive T cells which respond to the same epitope. The presence of Tregs in the TME is widely studied, leading to varying conclusions regarding their influence on the anti-tumour immune response (DeLeew et al. 2012). The diversity of these outcomes can be due to differences in tumours studied. However, it can also be due to usage of FoxP3 as the marker to identify/select Tregs, since the expression of this molecule is not fully restricted to Tregs (Fridman et al. 2012). In a large meta-analysis over 16 cancer types, DeLeew et al. found that in colorectal cancer, FoxP3+ cells were associated with good prognosis in four studies and neutral in six, whilst in melanoma, two studies found a correlation with poor prognosis and two papers reported a neutral association. Several factors were addressed that could possibly be of influence, like cancer grade or severity, presence of other lymphocytes, and even publication bias. Nevertheless, the prognostic value of FoxP3+ T cell infiltration was found to depend foremost on the specific type of cancer (DeLeew et al. 2012). The complexity of the influence of tumour-infiltrating FoxP3+ cells is also illustrated by a recent study: Two phenotypically distinct subpopulations of these lymphocytes had a different effect on colon cancer prognosis (Saito et al. 2016). While one population of FoxP3+ cells was highly suppressive Tregs, the other did not have this effect and instead secreted pro-inflammatory cytokines. An indication of the presence of primarily FoxP3+ non-Tregs in a tumour is increased expression of IL-12 and TGF-β. In tumours with effective Tregs, high FoxP3+ expression is associated with poor prognosis, whilst in tumours with non-Tregs, this was not the case (Saito et al. 2016).

## Cytokine patterns in cancer

Cytokines are well known for their regulatory role in immune responses and signalling between immune cells; however, they also play a role in cancer. An extensive review published by Lippitz describes cytokine patterns in cancer in an effort to elucidate the underlying immune responses. The emerging pattern is one of simultaneous immune stimulation and suppression, which holds up in spite of heterogeneity within the tumour and between different types of cancer (Lippitz 2013).

Pro-inflammatory cytokines secreted by TH1 cells, like IL-2, IL-12 and IFN-γ, are associated with good prognosis (Fridman et al. 2012; Mantovani et al. 2008; Lippitz 2013). In contrast, immunosuppressive cytokines are associated with poor outcome, especially IL-6, IL-10 and TGF-β (Lippitz 2013). These can be secreted by inhibitory cells like Tregs and also by tumour cells or surrounding tissue (Vesely et al. 2011).

Overall, cytokine profiles in cancer patients show reduced levels of pro-inflammatory cytokines and increased levels of immunosuppressive cytokines compared to healthy individuals (Whiteside 2013). Especially the role of IL-6, a multifunctional cytokine, with cancer, has been strongly established (Guo et al. 2012). Increased serum concentrations of IL-6 were found in at least 13 types of cancer, and associated with advanced disease and poor prognosis (Lippitz 2013). The signalling pathway via which IL-6 interferes is through activation of the signal transducer and transcription activator 3 (STAT3), which stimulates proliferation and has anti-apoptotic effects (Guo et al. 2012). Furthermore, IL-6 induces the production of matrix metalloproteinases by fibroblasts (Lippitz 2013), which can degrade the extracellular matrix and are likely to be involved in tissue evasion and metastasis (Hanahan and Weinberg 2011).

IL-2 serves as growth signal for CTLs and therefore is an important pro-inflammatory cytokine for anti-tumour responses. Without IL-2 signalling CD8+, T cells render anergic upon stimulation of their TCR (Ahrends and Borst 2018). IL-12 and IFN-γ are the other two main cytokines produced by TH1 cells. They are linked with each other in a positive feedback loop via antigen-presenting cells (APCs) (Lippitz 2013). IFN-γ stimulates the presentation of peptides in context of MHC class-II molecules, which is necessary to activate CD4+ T lymphocytes. In turn, IFN-γ production is stimulated by IL-12 which is produced by APCs (DCs or macrophages during antigen presentation), by NK cells in early response to infection and later on by CTLs. In cancer, this feedback loop is deregulated, leading to a decrease in IL-12 and IFN-γ. Due to its importance in antigen presentation, decreased levels of IFN-γ probably make a tumour less immunogenic (Lippitz 2013). Sufficient (or “functional”) concentrations of IL-2, IL-12 and IFN-γ were positively associated with prognosis in cancer (Fridman et al. 2012).

Inhibitory cytokines like TGF-β and IL-10 have similar positive feedback loops to maintain an immunosuppressive state (Lippitz 2013). TGF-β suppresses the production of IFN-γ, IL-12 and IL-2, in addition to inhibiting proliferation of T cells due to lack of IL-2 stimulation. A general finding amongst cancer types is that increased TGF-β is associated with worse prognosis (Lippitz 2013). Tumour cells are found to produce TGF-β after immunological escape (Vesely et al. 2011). Similarly, IL-10 inhibits pro-inflammatory cytokines, together with T cell activation and differentiation due to downregulation of MHC class II and the DC co-stimulatory ligands CD80/86 which stimulate CTL maturation. Tregs produce IL-10 and TGF-β; the latter is also necessary for both Treg and TH17 differentiation (Bettelli et al. 2006).

Chemotactic cytokines or chemokines regulate the migration of immune cells. Together with adhesive molecules, chemokines are, therefore, responsible for tumour infiltration of specific lymphocyte populations (Chen and Mellman 2013). A study in colorectal cancer showed that chemokines CXCL9 and CXCL10 attract memory T lymphocytes and CX3CL1 recruits CTLs towards the tumour (Mlecnik et al. 2010). These chemokines are associated with increased numbers of CTLs infiltrating the tumour and longer disease-free survival. Other general inflammatory chemokines like CCL3 and CCL5 can also recruit T lymphocytes towards the tumour (Franciszkiewicz et al. 2012). They are associated with improved outcome in lung cancer, but with poor prognosis in breast cancer. This differential effect can be explained by the recruitment of immune cells other than CTLs, like Tregs, TH17 cells and MDSCs. In addition to lymphocyte migration, chemokines also play a role in shaping the phenotype of T cells, e.g., through increased T cell activation by improved binding to antigen presenting cells such as DCs. Ligands for CCR7, CCR5 and CXCR4 are known to have this effect (Franciszkiewicz et al. 2012). Although promising, further research is necessary before prognostic value can be attributed to these chemokines.

## Infiltration of other immune cells

The TME is also infiltrated by innate immune cells, like macrophages, NK cells and myeloid-derived suppressor cells (MDSCs) (Angelova et al. 2015). Pro-inflammatory M1 macrophages are associated with anti-tumour responses and overexpress transcription factor NF-κB, whilst M2 macrophages have an inhibited NF-κB pathway and are associated with blood vessel formation and tumour growth (Mantovani et al. 2008). Myeloid-derived suppressor cells (MDSCs) form a heterogeneous group which can inhibit the functioning of CTLs and other immune cells in various ways (Fridman et al. 2012), e.g., it has been shown that reducing their numbers can enhance CTL response (Alizadeh et al. 2014). NK cells are important for clearing aberrant cells early in carcinogenesis if they lose MHC expression (Khong and Restifo 2002); however, tumour cells can become invisible for NK cells due to the loss of ligands for which NK cells have specific receptors (Salih et al. 2002). Therefore, the contribution of NK cells seems to be limited for controlling established tumours. A study in mice showed that the tumour is controlled despite treatment with antibodies against NK cells, receptors or effector molecules (Koebel et al. 2007).

Ratios between the amounts of immunostimulatory/−inhibitory cells and effector molecules can be more important than merely their presence. Davoli et al. associate somatic copy number alterations (SCNAs) with gene expression of immune markers in 12 cancer types (Davoli et al. 2017). Elevated ratios of pro/anti-inflammatory cytokines, M1/M2 macrophages and CTLs/Tregs are used as indication of an anti-tumour response. High levels of focal SCNAs, < 50% of a chromosome arm, were found to be associated with lower ratios and a shift towards immune escape across the different cancer types, with the exception of brain tumours. Low SCNA levels correlated with long-term survival after anti-CTLA-4 immunotherapy for melanoma and were better for predicting survival than the number of mutations (Davoli et al. 2017). Davoli et al. propose SCNA level as biomarker for immunotherapy; however, a biological mechanistic explanation is yet lacking, and validation of this biomarker is difficult due to the use of novel indicators like “SCNA level” which can differ a lot between datasets due to the intensive normalisation during its calculation.

## Influences on the efficacy of the CTL phenotype

The phenotype of cytotoxic T cells can be characterised by the secretion of effector molecules upon binding their cognate antigen. The fraction of tumour-specific CTLs producing IFN-γ correlates positively with treatment outcome (Schaefer et al. 2012). Priming and activation of CTLs by dendritic cells (DCs) are important for acquiring a good effector phenotype (Van De Ven and Borst 2015). The presence of other T cells influences the interaction between DCs and CTLs in the lymph node, as well as in the TME. Help from tumour-specific CD4+ cells is often necessary for DC activation and upregulation of co-stimulatory ligands, as innate immune receptors on DCs are often not sufficiently activated by tumour material (Van De Ven and Borst 2015). On the other hand, Tregs carry receptors like CTLA-4 which sequesters DC ligands CD80/86, resulting in less co-stimulation for cytotoxic T cells. At the moment, we are lacking a biomarker that can indicate whether T cell priming by DCs in tumour-draining lymph nodes forms a bottleneck for treatment response to ACT.

Treatment with an antibody which can stimulate CD27 can be an alternative way to improve the CTL effector phenotype (Van De Ven and Borst 2015). CD27 agonism can help to overcome a lack of co-stimulation by DCs and promote the effector differentiation of both CTLs and TH1 cells. Anti-CTLA-4 antibodies are already used to improve co-stimulation via the CD28 receptor by preventing the sequestering of its ligand by CTLA-4 (Snyder et al. 2014). A CD27 agonist could possibly replace anti-CTLA-4 and be used in conjunction with PD-1/PD-L1 antagonists. Antibodies against PD-1 or PD-L1 were developed since the activation of this pathway was discovered to be very immunosuppressive for cytotoxic T cells (Rizvi et al. 2015; Topalian et al. 2012). PD-L1 is often present on Tregs and can be expressed by tumour cells in late stages of cancer (Vesely et al. 2011). Combining a CD27 agonist with PD-1/PD-L1 antagonists would target both the bottleneck formed by peripheral tolerance and immunosuppression in the tumour microenvironment (Van De Ven and Borst 2015).

Tumour metabolism also affects T cell effector function, especially increased glycolysis could be associated with decreased anti-tumour immune responses (Cascone et al. 2018). Upregulation of glycolysis pathway genes was linked to decreased T cell attraction through CXCL10, less tumour infiltration, and an impaired CTL effector phenotype in melanoma and NSCLC (Cascone et al. 2018). Glycolysis pathway gene expression and the presence of lactic acid in the TME are promising biomarkers for ACT. Because melanoma tumours which were not responsive to ACT showed upregulation of glycolytic activity, and lactic acid inhibits TIL activity in a dose-dependant manner. Luckily, lactic acid inhibitors could restore the effector phenotype of CTLs (Cascone et al. 2018).

## Biomarkers in the tumour microenvironment

In order to assess whether the TME forms a bottleneck for ACT and requires additional interventions, the balance between known stimulatory and inhibitory factors should be considered.

There are several TME characteristics that can have prognostic value for ACT. Pro-inflammatory cytokines IL-2, IL-12 and IFN-γ are associated with good prognosis in cancer, whilst an increase of immunosuppressive cytokines IL-6, IL-10 and TGF-β correlates to poor outcome (Lippitz 2013; Fridman et al. 2012; Mantovani et al. 2008; Guo et al. 2012; Vesely et al. 2011). For infiltrating innate immune cells, a similar pattern can be observed regarding the effect on anti-tumour immune response: Pro-inflammatory M1 macrophages are beneficial, whilst M2s and MDSCs are associated with a poor outcome (Fridman et al. 2012; Mantovani et al. 2008). The contribution of tumour infiltrating NK cells in controlling tumour growth seems to be limited (Koebel et al. 2007, see above). For other TILs such as TH17, B cells and Tregs, their prognostic value is not yet established due to contradictory findings (DeLeew et al. 2012; Wilke et al. 2011; Saito et al. 2016). These factors should be considered per patient and per cancer type in order to assess whether the tumour microenvironment forms a problem for ACT and requires additional interventions.

Other promising biomarkers for ACT are increased glycolysis pathway gene expression and lactic acid in the TME, which both seem to down regulate immune responses (Cascone et al. 2018). At the moment, it is not yet known whether T cell priming by DCs in tumour-draining lymph nodes forms a bottleneck for treatment response to ACT. Screening CTLs for INF-y secretion as response to tumour-specific antigens seems to be a solid indicator of an effector phenotype (Schaefer et al. 2012). If this is found to be impaired, additional interventions could be necessary.

## Discussion and conclusion

All the biomarkers we discussed so far are studied in depth to identify their associations with cancer immunotherapies. Which biomarker is then the best one to use for ACT? None of the candidates seem to have the perfect predictive value. Therefore, we propose to combine various of these biomarkers as they are shown to have prognostic value for other cancer immunotherapies. In addition, they reflect the steps necessary to establish an adequate anti-tumour response, and fit well in the context of the cancer immunity cycle model (Chen and Mellman 2013). If a part of this cycle is suppressed or disrupted, the anti-tumour immune activity is endangered. The most researched bottlenecks are (1) recognition of the tumour, (2) priming and activation of tumour-specific T cells, and (3) immunosuppression in the tumour micro-environment (Van De Ven and Borst 2015). Characteristics indicative of these blockades are likely to be useful biomarkers for the efficacy of ACT, as well as other immunotherapies.

For the first bottleneck, recognition of the tumour, neoantigen presentation is a good candidate biomarker for ACT, especially when within-tumour heterogeneity is taken into account. WES of multiple regions can be combined with immunogenicity predictions to identify neoantigens expressed in the context of the patient’s HLA genotype and their distribution throughout the tumour (van Rooij et al. 2013; Marty et al. 2017). It is important to target multiple neoantigens with ACT in order to prevent immune escape by losing the presentation of these neoantigens (Anagnostou et al. 2017). Heterogeneous tumours have more potential for immune escape, whilst more homogeneous tumours are associated with more immune cell infiltration and better prognosis (Morgan et al. 2013; Angelova et al. 2015). Especially tumours with a large number of clonal neoantigens were found to respond well to immunotherapy (McGranahan et al. 2016). Immune escape can occur through loss of cell-surface presentation of immunogenic neoantigens, e.g., as result of losing the mutation itself (Anagnostou et al. 2017) or via downregulation of MHC-I. Hence, it is not surprising that for cancers with a moderate mutational load, the number of mutations only correlated with improved immune response when combined with MHC-I expression (Brown et al. 2014; Matsushita et al. 2016). Additionally, decreased expression of MHC-I was found to be associated with a poor prognosis (Perea et al. 2017; Mariya et al. 2014; Zeestraten et al. 2014).

For tumours with little or highly heterogeneous neoantigen presentation, ACT seems to be a less suitable treatment option because of the first bottleneck. An alternative is to engineer CTLs with high-avidity TCRs specific for TAAs. These cells are shown to elicit anti-tumour responses in clinical trials; however, targeting overexpressed or cancer-germline antigens sometimes resulted in severe adverse effects (Parkhurst et al. 2011; Morgan et al. 2013; Robbins et al. 2015). For this reason, we would advise against targeting TAAs with engineered TCRs, as it is impossible to precisely characterise the tissue distribution of these antigens. In addition, at least for colon cancer, it is speculated that immune responses are primarily directed against neoantigens and not cancer-germline antigens (Angelova et al. 2015).

Another important factor to consider for ACT is the number and diversity of tumour-specific CTLs that can be administered. If neoantigens could be identified, subsequently screening measures like MHC tetramers or neoantigen-pulsed APCs can be used to check for the presence of tumour-specific T cells (Schumacher and Schreiber 2015). However, this is often a limiting factor when insufficient cells are isolated from resected tumour tissue, because acquiring them from peripheral blood will be even more problematic as the fraction is much lower (Schumacher, personal communication). Fortunately, advances have recently been made in isolating tumour-specific CTLs from peripheral blood with an effective phenotype (Cohen et al. 2015; Gros et al. 2016).

The second bottleneck, the priming and activation of the tumour-specific CTLs, has to be overcome to allow CTLs to infiltrate the tumour and have a good effector phenotype for controlling the tumour. Hereto, priming and activation of CTLs by dendritic cells DCs are important (Van De Ven and Borst 2015). To our knowledge, there is not yet a biomarker that can indicate whether T cell priming and activation form a bottleneck for treatment response to ACT. Nevertheless, the immunoscore is proposed as measure for tumour infiltration by T cells and prognostic biomarker for immunotherapy (Galon et al. 2012). It has been found useful for a.o. colorectal cancer, gastric cancer and melanoma (Pagès et al. 2018; Jiang et al. 2018; Galon et al. 2017), and therefore, can function as a possible biomarker for ACT success. Additionally, CTL effector phenotypes can be assessed by interferon-γ ELISPOT since it can measure how responsive CTLs are to specific epitopes (Schaefer et al. 2012).

The third bottleneck, the immunosuppression by other immune cells and cytokines in TME, can inhibit success of an ACT even if adequate tumour recognition and infiltration is secured. One promising biomarker for this bottleneck is increased glycolysis in the TME, which is associated with decreased CTL activity and a decreased immune response (Cascone et al. 2018). In general, a tendency towards pro-inflammatory cytokines and innate immune cells is associated with good prognosis, whilst immunosuppressive factors can be linked to poor outcome (Lippitz 2013; Fridman et al. 2012). Noteworthy is that the prognostic value of Tregs seems to differ between cancer types (DeLeew et al. 2012; Saito et al. 2016). For immune checkpoint blockade, peripheral immunological signatures have been proposed as biomarker of suppression (Martens et al. 2016; Tanizaki et al. 2018). However, it is unclear how these would relate to efficacy of ACT, since they are rather specific for the biological mechanisms behind the checkpoint blockade therapies. It is especially important to consider each patient case separately, and decide if additional interventions are necessary to improve the response to ACT.

Concluding, good candidates for adoptive cell transfer are tumours that have a high clonal neoantigen burden and for which a diverse tumour-specific CTL population can be isolated. Furthermore, these tumour-specific CTLs should have a good effector phenotype and the TME should be biased towards immunostimulatory factors. Presentation of neoantigens covering (almost) the entire tumour can be considered the most strict criterion, since this cannot be replaced by or improved with additional treatment (Fig. 1).

This review is limited to application of adoptive cell transfer to treat solid tumours with the patient’s cytotoxic CD8+ T cells. However, ACT can also be combined with other immunotherapies or conducted with different types of immune cells, for example CD4+ cells (Friedman et al. 2012).

Using a combination of different biomarkers is advisable, especially because of the complexity of immunological interactions. Before the biomarkers discussed in this review can be used as biomarker, they need to be validated for specific tumour types and in multiple large patient cohorts to overcome the diversity caused by intrinsic differences between tissues that contain the tumours and their interactions with the immune system. In addition, for a biomarker to be useful in a therapeutic setting, it is important that assays are standardised, reproducible and fast (Willis and Lord 2015). Advances in neoantigen identification such as described by Marty et al. 2017, and isolation of CTLs responding to those antigens with a high-throughput assay like the MHC multimers (Hadrup et al. 2009) are especially important. Further improvements in techniques to characterise (i) the tumour environment via biopsy or after resection, (ii) the tumour heterogeneity, and (iii) intra-tumoral selection pressure towards immune evasion are all required to improve the efficacy of immunotherapy and to predict a patient’s response to ACT.
